# 1-Benzyl-2,5-dioxopyrrolidine-3,4-diyl diacetate

**DOI:** 10.1107/S1600536810043187

**Published:** 2010-11-06

**Authors:** Ignez Caracelli, Fernando P. Ferreira, Adriano S. Vieira, Hélio A. Stefani, Carlos A. De Simone, Edward R. T. Tiekink

**Affiliations:** a Physics Department, Universidade Federal de São Carlos, 13565-905 São Carlos, SP, Brazil; b Departamento de Farmácia, Faculdade de Ciências Farmacêuticas, Universidade de São Paulo, São Paulo, SP, Brazil; c Instituto de Química e Biotecnologia, Universidade Federal de Alagoas, 57072-970 Maceió, AL, Brazil; d Department of Chemistry, University of Malaya, 50603 Kuala Lumpur, Malaysia

## Abstract

The pyrrolidine-2,5-dione ring in the title compound, C_15_H_15_NO_6_, is in a twisted conformation with the acetyl C atoms projecting to opposite sides of the ring. The acetyl groups lie to opposite sides of the five-membered ring. The benzene ring is roughly perpendicular to the heterocyclic ring, forming a dihedral angle of 76.57 (14)° with it. In the crystal, mol­ecules are connected through a network of C—H⋯O and C—H⋯π inter­actions.

## Related literature

For the use of *N*-acyl­iminium in organic synthesis, see: Vieira *et al.* (2008 ▶); Huang (2006 ▶); Russo *et al.* (2010 ▶). For background to the synthesis, see: Caracelli *et al.* (2010 ▶). For a related structure, see: Naz *et al.* (2009 ▶). For conformational analysis, see: Cremer & Pople (1975 ▶); Iulek & Zukerman-Schpector (1997 ▶).
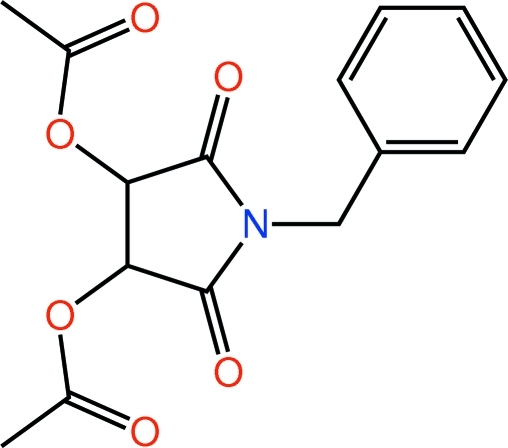


## Experimental

### 

#### Crystal data

C_15_H_15_NO_6_
*M* *_r_* = 305.28
Orthorhombic, 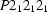

*a* = 8.8498 (4) Å
*b* = 9.8107 (4) Å
*c* = 17.5148 (6) Å
*V* = 1520.68 (11) Å^3^
*Z* = 4
Mo *K*α radiation
μ = 0.10 mm^−1^
*T* = 290 K
0.28 × 0.23 × 0.06 mm

#### Data collection

Bruker SMART APEXII diffractometer
Absorption correction: multi-scan (*SADABS*; Sheldrick, 1996 ▶) *T*_min_ = 0.962, *T*_max_ = 0.991
7672 measured reflections
1541 independent reflections
1396 reflections with *I* > 2σ(*I*)
*R*_int_ = 0.122

#### Refinement

*R*[*F* ^2^ > 2σ(*F* ^2^)] = 0.036
*wR*(*F* ^2^) = 0.085
*S* = 1.07
1541 reflections
202 parameters
H-atom parameters constrained
Δρ_max_ = 0.12 e Å^−3^
Δρ_min_ = −0.12 e Å^−3^

### 

Data collection: *APEX2* (Bruker, 2007 ▶); cell refinement: *SAINT* (Bruker, 2007 ▶); data reduction: *SAINT*; program(s) used to solve structure: *SIR97* (Altomare *et al.*, 1999 ▶); program(s) used to refine structure: *SHELXL97* (Sheldrick, 2008 ▶); molecular graphics: *ORTEP-3* (Farrugia, 1997 ▶) and *DIAMOND* (Brandenburg, 2006 ▶); software used to prepare material for publication: *publCIF* (Westrip, 2010 ▶).

## Supplementary Material

Crystal structure: contains datablocks global, I. DOI: 10.1107/S1600536810043187/hg2732sup1.cif

Structure factors: contains datablocks I. DOI: 10.1107/S1600536810043187/hg2732Isup2.hkl

Additional supplementary materials:  crystallographic information; 3D view; checkCIF report

## Figures and Tables

**Table 1:** Hydrogen-bond geometry (Å, °)

*D*—H⋯*A*	*D*—H	H⋯*A*	*D*⋯*A*	*D*—H⋯*A*
C2—H2⋯O3^i^	0.98	2.51	3.206 (2)	128
C3—H3⋯O1^ii^	0.98	2.55	3.276 (3)	131
C5—H5b⋯O6^iii^	0.97	2.49	3.328 (3)	144
C13—H13b⋯*Cg*1^ii^	0.96	2.95	3.702 (3)	136

Symmetry codes: (i) 
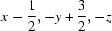
; (ii) 
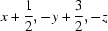
; (iii) 
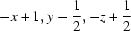
. *Cg*1 is the centroid of the C6–C11 ring.

**Fig. 1.**
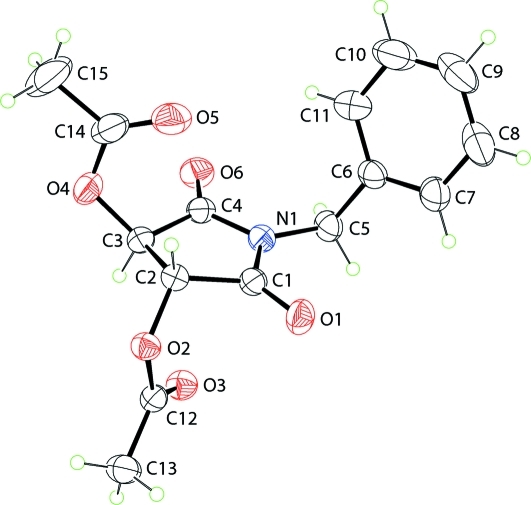
Molecular structure of (I) showing atom labelling scheme and displacement ellipsoids at the 35% probability level.

**Fig. 2.**
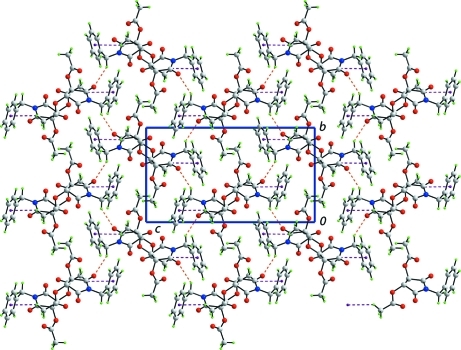
Unit-cell contents shown in projection down the a axis in (I). The C—H···O and C—H···π contacts are shown as orange and purple dashed lines, respectively.

## supplementary crystallographic information

### Comment

*N*-Acyliminium ions are very important in organic synthesis since they are reactive intermediates involved in the synthesis of many compounds with interesting biological properties (Vieira *et al.*, 2008). Nucleophilic additions to *N*-acyliminium ions constitute an important method to provide α-functionalized amino compounds and for the preparation of alkaloids (Huang, 2006) and many other biologically active nitrogen heterocycles, such as ethosuximide used in the treatment of epilepsy (Russo *et al.*, 2010). As part of our on-going research interest in bioactive compounds (Caracelli *et al.*, 2010) the title compound, (I), was synthesized and its crystal structure determined as described herein.

The molecular structure of (I), Fig. 1, shows the pyrrolidine-2,5-dione ring [r.m.s. deviation of the N1,C1–C4 atoms = 0.105 Å] to adopt a twisted conformation with the C2 and C3 atoms being displaced by 0.089 (2) and -0.087 (2) Å, respectively, out of the plane. The ring-puckering parameters are q_2_ = 0.149 (2) Å, and φ_2_ = 87.4 (8) ° (Cremer & Pople, 1975; Iulek & Zukerman-Schpector, 1997). The O1 and O6 atoms lie -0.178 (2) and 0.139 (2) Å out of the plane through the pyrrolidine ring, and the benzene ring is orientated to be normal to the ring as seen in the dihedral angle formed between their least-squares planes [76.57 (14) °]. The acetyl groups lie on opposite sides of the pyrrolidine plane. To a first approximation, the structure of (I) resembles that reported recently for the *N*-(*m*-tolyl) analogue (Naz *et al.*, 2009).

In the crystal packing, molecules are connected *via* C—H···O and C—H···π interactions, Fig. 2 and Table 1.

### Experimental

A mixture of *L*-tartaric acid (15.0 g, 100 mmol) and acetylchloride (70 ml, 1.0 mol) was stirred under reflux for 24 h under nitrogen atmosphere, during which the solution became homogeneous. Excess acetyl chloride was removed by distillation at 1 atm and trace amounts were removed under vacuum. The resulting crude anhydride was dissolved in dry THF (120 ml) and benzylamine (10.7 g,100 mmol) was slowly added. The solution was stirred for 4 h, and then concentrated in vacuum. The residue was then refluxed with acetyl chloride (70 ml, 1.0 mol) for another 5 h. After concentration of the reaction mixture under vacuum, the residue was purified by column chromatography (*n*-hexane/ethyl acetate, 2:1) to give the title compound (23.8 g, 78%) as a white solid. Single crystals of (I) were obtained by slow evaporation from its ethyl acetate-hexane solution.

### Refinement

Carbon-bound H-atoms were placed in calculated positions (C–H = 0.93 to 0.98 Å) and were included in the refinement in the riding model approximation, with *U*_iso_(H) set to 1.2–1.5*U*_equiv_(C). In the absence of significant anomalous scattering effects, 1119 Friedel pairs were averaged in the final refinement. However, the absolute configuration was assigned on the basis of the chirality of the *L*-tartaric acid starting material.

### Crystal data

**Table:**

C_15_H_15_NO_6_	*F*(000) = 640
*M**_r_* = 305.28	*D*_x_ = 1.333 Mg m^−^^3^
Orthorhombic, *P*2_1_2_1_2_1_	Mo *K*α radiation, λ = 0.71073 Å
Hall symbol: P 2ac 2ab	Cell parameters from 5560 reflections
*a* = 8.8498 (4) Å	θ = 2.9–26.4°
*b* = 9.8107 (4) Å	µ = 0.10 mm^−^^1^
*c* = 17.5148 (6) Å	*T* = 290 K
*V* = 1520.68 (11) Å^3^	Block, colourless
*Z* = 4	0.28 × 0.23 × 0.06 mm

### Data collection

**Table:**

Bruker SMART APEXII diffractometer	1541 independent reflections
Radiation source: fine-focus sealed tube	1396 reflections with *I* > 2σ(*I*)
graphite	*R*_int_ = 0.122
φ and ω scans	θ_max_ = 25.0°, θ_min_ = 3.1°
Absorption correction: multi-scan (*SADABS*; Sheldrick, 1996)	*h* = −10→10
*T*_min_ = 0.962, *T*_max_ = 0.991	*k* = −10→11
7672 measured reflections	*l* = −20→20

### Refinement

**Table:**

Refinement on *F*^2^	Secondary atom site location: difference Fourier map
Least-squares matrix: full	Hydrogen site location: inferred from neighbouring sites
*R*[*F*^2^ > 2σ(*F*^2^)] = 0.036	H-atom parameters constrained
*wR*(*F*^2^) = 0.085	*w* = 1/[σ^2^(*F*_o_^2^) + (0.0293*P*)^2^ + 0.1923*P*] where *P* = (*F*_o_^2^ + 2*F*_c_^2^)/3
*S* = 1.07	(Δ/σ)_max_ = 0.001
1541 reflections	Δρ_max_ = 0.12 e Å^−^^3^
202 parameters	Δρ_min_ = −0.12 e Å^−^^3^
0 restraints	Extinction correction: *SHELXL97* (Sheldrick, 2008), Fc^*^=kFc[1+0.001xFc^2^λ^3^/sin(2θ)]^-1/4^
Primary atom site location: structure-invariant direct methods	Extinction coefficient: 0.111 (10)

### Special details

**Table:**

Geometry. All s.u.'s (except the s.u. in the dihedral angle between two l.s. planes) are estimated using the full covariance matrix. The cell s.u.'s are taken into account individually in the estimation of s.u.'s in distances, angles and torsion angles; correlations between s.u.'s in cell parameters are only used when they are defined by crystal symmetry. An approximate (isotropic) treatment of cell s.u.'s is used for estimating s.u.'s involving l.s. planes.
Refinement. Refinement of *F*^2^ against ALL reflections. The weighted *R*-factor *wR* and goodness of fit *S* are based on *F*^2^, conventional *R*-factors *R* are based on *F*, with *F* set to zero for negative *F*^2^. The threshold expression of *F*^2^ > 2σ(*F*^2^) is used only for calculating *R*-factors(gt) *etc*. and is not relevant to the choice of reflections for refinement. *R*-factors based on *F*^2^ are statistically about twice as large as those based on *F*, and *R*- factors based on ALL data will be even larger.

### Fractional atomic coordinates and isotropic or equivalent isotropic displacement parameters (Å^2^)

**Table:**

	*x*	*y*	*z*	*U*_iso_*/*U*_eq_	
O1	0.25814 (16)	0.67116 (18)	0.09591 (10)	0.0555 (5)	
O2	0.42326 (15)	0.75768 (16)	−0.03939 (9)	0.0425 (4)	
O3	0.57172 (17)	0.60872 (18)	0.02191 (10)	0.0505 (4)	
O4	0.5859 (2)	1.03936 (19)	0.03516 (11)	0.0652 (6)	
O5	0.3864 (3)	1.1015 (2)	0.10556 (14)	0.0772 (6)	
O6	0.6412 (2)	0.93139 (18)	0.18931 (10)	0.0603 (5)	
N1	0.44087 (19)	0.79166 (19)	0.15977 (11)	0.0418 (5)	
C1	0.3594 (2)	0.7537 (2)	0.09654 (13)	0.0415 (5)	
C2	0.4146 (2)	0.8367 (2)	0.02866 (13)	0.0400 (5)	
H2	0.3419	0.9104	0.0199	0.048*	
C3	0.5611 (2)	0.9008 (2)	0.05719 (14)	0.0448 (5)	
H3	0.6453	0.8463	0.0374	0.054*	
C4	0.5564 (2)	0.8820 (2)	0.14324 (14)	0.0437 (5)	
C5	0.4073 (3)	0.7405 (3)	0.23638 (14)	0.0510 (6)	
H5A	0.4951	0.7538	0.2687	0.061*	
H5B	0.3875	0.6434	0.2336	0.061*	
C6	0.2737 (3)	0.8104 (2)	0.27199 (13)	0.0482 (6)	
C7	0.1408 (3)	0.7410 (3)	0.28440 (16)	0.0629 (7)	
H7	0.1336	0.6495	0.2708	0.075*	
C8	0.0181 (4)	0.8059 (4)	0.3169 (2)	0.0835 (10)	
H8	−0.0709	0.7579	0.3254	0.100*	
C9	0.0272 (5)	0.9408 (4)	0.3365 (2)	0.0896 (12)	
H9	−0.0554	0.9845	0.3584	0.108*	
C10	0.1573 (5)	1.0103 (4)	0.3238 (2)	0.0956 (12)	
H10	0.1636	1.1019	0.3370	0.115*	
C11	0.2808 (4)	0.9457 (3)	0.2914 (2)	0.0748 (9)	
H11	0.3693	0.9944	0.2828	0.090*	
C12	0.5071 (2)	0.6412 (2)	−0.03556 (14)	0.0425 (5)	
C13	0.5040 (3)	0.5656 (3)	−0.10878 (16)	0.0586 (7)	
H13A	0.5864	0.5020	−0.1102	0.088*	
H13B	0.5135	0.6287	−0.1504	0.088*	
H13C	0.4102	0.5172	−0.1132	0.088*	
C14	0.4873 (5)	1.1318 (3)	0.06372 (18)	0.0722 (9)	
C15	0.5247 (7)	1.2725 (3)	0.0367 (3)	0.1274 (19)	
H15A	0.4450	1.3338	0.0509	0.191*	
H15B	0.5355	1.2721	−0.0178	0.191*	
H15C	0.6177	1.3018	0.0597	0.191*	

### Atomic displacement parameters (Å^2^)

**Table:**

	*U*^11^	*U*^22^	*U*^33^	*U*^12^	*U*^13^	*U*^23^
O1	0.0492 (8)	0.0622 (11)	0.0550 (11)	−0.0163 (8)	0.0025 (7)	0.0034 (9)
O2	0.0459 (7)	0.0426 (8)	0.0388 (8)	0.0016 (7)	−0.0020 (6)	−0.0024 (7)
O3	0.0532 (8)	0.0470 (9)	0.0514 (10)	0.0047 (8)	−0.0038 (8)	0.0011 (8)
O4	0.0894 (13)	0.0503 (10)	0.0558 (11)	−0.0256 (10)	0.0064 (10)	0.0006 (9)
O5	0.1066 (15)	0.0511 (11)	0.0739 (14)	0.0153 (12)	0.0061 (13)	−0.0027 (11)
O6	0.0602 (10)	0.0644 (11)	0.0562 (11)	−0.0106 (9)	−0.0110 (8)	−0.0119 (10)
N1	0.0427 (9)	0.0433 (10)	0.0395 (10)	0.0004 (8)	−0.0002 (7)	−0.0005 (9)
C1	0.0390 (10)	0.0409 (11)	0.0445 (12)	0.0007 (10)	0.0029 (9)	−0.0015 (11)
C2	0.0417 (10)	0.0394 (11)	0.0390 (11)	0.0005 (9)	0.0015 (9)	−0.0021 (10)
C3	0.0473 (11)	0.0414 (11)	0.0457 (13)	−0.0088 (10)	0.0046 (10)	−0.0064 (11)
C4	0.0438 (11)	0.0408 (12)	0.0465 (12)	0.0000 (11)	−0.0002 (10)	−0.0050 (10)
C5	0.0585 (12)	0.0517 (13)	0.0428 (12)	0.0063 (12)	0.0029 (10)	0.0077 (12)
C6	0.0628 (13)	0.0482 (12)	0.0337 (12)	0.0064 (12)	0.0026 (10)	0.0025 (11)
C7	0.0716 (15)	0.0587 (15)	0.0584 (17)	0.0038 (15)	0.0144 (13)	0.0088 (15)
C8	0.0765 (18)	0.101 (3)	0.073 (2)	0.016 (2)	0.0284 (16)	0.018 (2)
C9	0.104 (3)	0.100 (3)	0.0645 (19)	0.048 (2)	0.0299 (18)	0.007 (2)
C10	0.134 (3)	0.071 (2)	0.082 (2)	0.024 (2)	0.022 (2)	−0.019 (2)
C11	0.0864 (19)	0.0607 (16)	0.077 (2)	0.0022 (17)	0.0113 (17)	−0.0182 (17)
C12	0.0421 (10)	0.0384 (12)	0.0469 (13)	−0.0025 (10)	0.0043 (10)	−0.0014 (10)
C13	0.0644 (14)	0.0558 (15)	0.0557 (15)	−0.0018 (14)	0.0033 (12)	−0.0154 (14)
C14	0.123 (3)	0.0413 (14)	0.0525 (16)	−0.0093 (17)	−0.0130 (19)	0.0001 (14)
C15	0.246 (6)	0.0468 (18)	0.090 (3)	−0.032 (3)	−0.023 (3)	0.0162 (19)

### Geometric parameters (Å, °)

**Table:**

O1—C1	1.208 (2)	C6—C11	1.372 (4)
O2—C12	1.365 (3)	C6—C7	1.376 (4)
O2—C2	1.424 (3)	C7—C8	1.382 (4)
O3—C12	1.201 (3)	C7—H7	0.9300
O4—C14	1.354 (4)	C8—C9	1.370 (5)
O4—C3	1.430 (3)	C8—H8	0.9300
O5—C14	1.193 (4)	C9—C10	1.356 (6)
O6—C4	1.204 (3)	C9—H9	0.9300
N1—C1	1.373 (3)	C10—C11	1.385 (5)
N1—C4	1.384 (3)	C10—H10	0.9300
N1—C5	1.463 (3)	C11—H11	0.9300
C1—C2	1.522 (3)	C12—C13	1.481 (3)
C2—C3	1.525 (3)	C13—H13A	0.9600
C2—H2	0.9800	C13—H13B	0.9600
C3—C4	1.519 (3)	C13—H13C	0.9600
C3—H3	0.9800	C14—C15	1.496 (4)
C5—C6	1.502 (3)	C15—H15A	0.9600
C5—H5A	0.9700	C15—H15B	0.9600
C5—H5B	0.9700	C15—H15C	0.9600
			
C12—O2—C2	116.37 (16)	C6—C7—H7	119.7
C14—O4—C3	116.0 (2)	C8—C7—H7	119.7
C1—N1—C4	113.15 (19)	C9—C8—C7	120.2 (4)
C1—N1—C5	122.70 (18)	C9—C8—H8	119.9
C4—N1—C5	124.15 (19)	C7—C8—H8	119.9
O1—C1—N1	125.4 (2)	C10—C9—C8	119.6 (3)
O1—C1—C2	126.2 (2)	C10—C9—H9	120.2
N1—C1—C2	108.44 (17)	C8—C9—H9	120.2
O2—C2—C1	112.34 (17)	C9—C10—C11	120.5 (3)
O2—C2—C3	116.94 (17)	C9—C10—H10	119.8
C1—C2—C3	103.76 (18)	C11—C10—H10	119.8
O2—C2—H2	107.8	C6—C11—C10	120.5 (3)
C1—C2—H2	107.8	C6—C11—H11	119.7
C3—C2—H2	107.8	C10—C11—H11	119.7
O4—C3—C4	112.8 (2)	O3—C12—O2	121.5 (2)
O4—C3—C2	115.7 (2)	O3—C12—C13	127.0 (2)
C4—C3—C2	104.60 (18)	O2—C12—C13	111.5 (2)
O4—C3—H3	107.8	C12—C13—H13A	109.5
C4—C3—H3	107.8	C12—C13—H13B	109.5
C2—C3—H3	107.8	H13A—C13—H13B	109.5
O6—C4—N1	125.3 (2)	C12—C13—H13C	109.5
O6—C4—C3	126.8 (2)	H13A—C13—H13C	109.5
N1—C4—C3	107.78 (18)	H13B—C13—H13C	109.5
N1—C5—C6	112.58 (19)	O5—C14—O4	122.9 (3)
N1—C5—H5A	109.1	O5—C14—C15	126.1 (4)
C6—C5—H5A	109.1	O4—C14—C15	111.0 (4)
N1—C5—H5B	109.1	C14—C15—H15A	109.5
C6—C5—H5B	109.1	C14—C15—H15B	109.5
H5A—C5—H5B	107.8	H15A—C15—H15B	109.5
C11—C6—C7	118.6 (3)	C14—C15—H15C	109.5
C11—C6—C5	120.5 (3)	H15A—C15—H15C	109.5
C7—C6—C5	120.8 (2)	H15B—C15—H15C	109.5
C6—C7—C8	120.6 (3)		
			
C4—N1—C1—O1	176.2 (2)	O4—C3—C4—O6	−44.1 (3)
C5—N1—C1—O1	−3.7 (3)	C2—C3—C4—O6	−170.7 (2)
C4—N1—C1—C2	−5.8 (2)	O4—C3—C4—N1	138.79 (19)
C5—N1—C1—C2	174.33 (19)	C2—C3—C4—N1	12.2 (2)
C12—O2—C2—C1	−54.5 (2)	C1—N1—C5—C6	−77.9 (3)
C12—O2—C2—C3	65.3 (2)	C4—N1—C5—C6	102.2 (2)
O1—C1—C2—O2	−41.8 (3)	N1—C5—C6—C11	−67.2 (3)
N1—C1—C2—O2	140.22 (17)	N1—C5—C6—C7	111.6 (3)
O1—C1—C2—C3	−169.0 (2)	C11—C6—C7—C8	−1.0 (5)
N1—C1—C2—C3	13.0 (2)	C5—C6—C7—C8	−179.7 (3)
C14—O4—C3—C4	−55.3 (3)	C6—C7—C8—C9	0.6 (5)
C14—O4—C3—C2	65.1 (3)	C7—C8—C9—C10	0.0 (6)
O2—C2—C3—O4	96.2 (2)	C8—C9—C10—C11	−0.2 (6)
C1—C2—C3—O4	−139.5 (2)	C7—C6—C11—C10	0.9 (5)
O2—C2—C3—C4	−139.1 (2)	C5—C6—C11—C10	179.6 (3)
C1—C2—C3—C4	−14.8 (2)	C9—C10—C11—C6	−0.3 (6)
C1—N1—C4—O6	178.6 (2)	C2—O2—C12—O3	−1.1 (3)
C5—N1—C4—O6	−1.5 (3)	C2—O2—C12—C13	178.27 (18)
C1—N1—C4—C3	−4.2 (2)	C3—O4—C14—O5	0.9 (4)
C5—N1—C4—C3	175.7 (2)	C3—O4—C14—C15	−179.9 (3)

### Hydrogen-bond geometry (Å, °)

**Table:**

*D*—H···*A*	*D*—H	H···*A*	*D*···*A*	*D*—H···*A*
C2—H2···O3^i^	0.98	2.51	3.206 (2)	128
C3—H3···O1^ii^	0.98	2.55	3.276 (3)	131
C5—H5b···O6^iii^	0.97	2.49	3.328 (3)	144
C13—H13b···Cg1^ii^	0.96	2.95	3.702 (3)	136

Symmetry codes: (i) *x*−1/2, −*y*+3/2, −*z*; (ii) *x*+1/2, −*y*+3/2, −*z*; (iii) −*x*+1, *y*−1/2, −*z*+1/2.
